# The Association Between the Occurrence of Sensory Integration Disorders, Depression, and Chronic Fatigue in Patients with Relapsing–Remitting Multiple Sclerosis

**DOI:** 10.3390/jcm15010065

**Published:** 2025-12-22

**Authors:** Karolina Machowska-Sempruch, Marta Masztalewicz, Agnieszka Meller, Przemysław Nowacki, Wioletta Pawlukowska

**Affiliations:** Department of Neurology, Pomeranian Medical University, 71252 Szczecin, Polandwioletta.pawlukowska@pum.edu.pl (W.P.)

**Keywords:** multiple sclerosis, sensory integration disorder, depression, fatigue, relapsing–remitting MS

## Abstract

**Background/Objectives:** Multiple sclerosis (MS) is a chronic neurological disease associated not only with motor dysfunction but also with non-motor symptoms such as depression and MS-related fatigue (MSRF). Sensory integration disorders (SID) in MS remain poorly characterized. This study aimed to evaluate the association between SID and depression, MSRF, disability level, disease duration, and disease activity in patients with relapsing–remitting MS (RRMS). **Methods:** A total of 205 patients with RRMS were assessed using the Daniel Travis sensory integration questionnaire, Beck Depression Inventory (BDI), EDSS, FSMC fatigue scale, and MRI T2-lesion burden. Due to non-normal distribution, non-parametric statistical tests were applied with significance set at *p* < 0.05. **Results:** Statistically significant associations were identified between SID and depressive symptoms. Compared with patients without depression, individuals with mild to severe depression showed significantly higher impairment in under-responsiveness/sensory seeking (*p* = 0.040), sensory discrimination (*p* = 0.017 for mild; *p* = 0.011 for severe), sensory-based motor abilities (*p* = 0.007 for severe), and social–emotional functioning (*p* = 0.006 for mild; *p* = 0.014 for severe). Higher disability (EDSS > 3) was associated with impaired sensory discrimination (*p* = 0.013) and reduced motor abilities (*p* = 0.000). Longer disease duration was linked to poorer general sensory modulation (≤5 vs. 5–10 years *p* = 0.038; ≤5 vs. >10 years *p* = 0.037) and reduced motor abilities (>10 years *p* = 0.042). Increased disease activity (1 or ≥2 relapses/year) correlated with more severe under-responsiveness/sensory seeking (*p* = 0.039) and worse social–emotional functioning (*p* = 0.025 and *p* = 0.007). No significant association was found between SID and MSRF or MRI T2-lesion count. **Conclusions:** In conclusion, sensory integration disorders in RRMS are strongly associated with depression, disability level, disease duration, and relapse rate, but not with MSRF. SID assessment may provide additional insight into non-motor symptom burden and disease progression.

## 1. Introduction

Multiple sclerosis (MS) is a chronic, incurable disease of the central nervous system, the direct cause of which remains unknown, although an autoimmune background is assumed. The disease has a varied course, but systematically leads to neurological deterioration and is the most common non-traumatic cause of disability in young adults. The disease process involves the formation of diffuse foci of demyelination, as well as degeneration of the white and gray matter of the central nervous system. Depending on the location of the foci of damage, a range of clinical manifestations are observed [1,2].

The relapsing–remitting form of MS (RRMS) accounts for approximately 85% of initial diagnoses. RRMS is defined by episodic neurological worsening (relapses) lasting at least 24 h, followed by periods of partial or complete recovery. These relapses reflect focal immune activation with breakdown of the blood–brain barrier, inflammatory demyelination, and axonal injury. Over time, many patients with RRMS accumulate residual deficits between relapses, and a proportion eventually transition into secondary progressive MS, characterized by gradual neurological decline independent of acute relapses. Despite significant therapeutic advances—including disease-modifying therapies (DMTs) targeting specific inflammatory pathways—RRMS remains heterogeneous in presentation and outcome, and many patients continue to experience long-term physical, sensory, and cognitive disability [1,2,3,4,5].

In addition to the symptoms of focal damage to the central nervous system in patients with MS, attention is also drawn to the high frequency of non-motor symptoms such as MS-related fatigue (MSRF), which can affect up to 80% of patients, depression (15–56% of patients in the later stages of the disease), cognitive dysfunction (11–67% of patients) [6].

Fatigue in MS is defined as a multifactorial pathology-both as a result of damage to the central nervous system in the course of MS, as well as a disorder caused by general weakness, sleep and mood disturbances, and side effects of the disease modyfying treatment used [7]. MSRF definitely negatively affects the quality of life of patients with MS [8].

Depressive disorders are present in approximately 50% of MS patients. They are two to three times more common in this group than in the general population. They negatively impact quality of life and treatment outcomes and become significantly more common as the disease progresses [9,10]. The pathophysiological basis of this process in MS is multifactorial, similar to that in MSRF. Neuroanatomical factors such as activation of hippocampal microglia, spreading demyelinating lesions, and atrophy of the cerebral cortex in strategic regions have been implicated, as well as adaptive factors resulting from the diagnosis of a severe, incurable, and potentially progressive degenerative disease [10]. Common mechanisms have been suggested to play a role in the development of both MSRF and depression, including psychological factors or brain damage in specific neuroanatomical pathways [10,11].

Recently, increasing importance has also been placed on somatosensory integration disorders [12,13,14].

The basis of somatosensory integration is that human motor and cognitive functioning is closely linked to the integration of stimuli from visual, auditory, tactile, olfactory, gustatory, vestibular and proprioceptive receptors. These stimuli are appropriately organized in the central nervous system and then used to formulate perception, learning, behavior, emotions, movement planning, muscle tension, among others.

Sensory integration dysfunctions influence the ability to organize sensory information, motor and cognitive performance.

One of the most well-established approaches to SID has been proposed by Winnie Dunn and her four-quadrant model of sensory processing [15].

According to this model, sensory processing consists of a neurological threshold (high or low) and a behavioral response.

A high neurological threshold refers to individuals who are hypersensitive and may represent two behavioral patterns, depending on their response strategy:

(1) Sensation-seeking—the individual represents an active response strategy and will seek out stimulus-rich environments to enhance the response;
(2) Low registration—the individual represents a passive response strategy and will show slow or no response to stimuli.

Low neurological threshold, on the other hand, refers to hypersensitivity and the following two behavioral patterns:

(3) Sensation avoidance—an active response strategy that results in the avoidance of sensations that are uncomfortable for the person;
(4) Sensitivity to sensations—a passive response strategy to sensations that may be unpleasant [15,16,17].

In both SID and MS, there may be some common symptoms, such as sensory abnormalities-central and musculoskeletal pain, limb paresthesias, allodynia (hypersensitivity in response to stimuli), dysesthesias, decreased vibration sensation or Lhermitte’s sign [12,18]. Sensory integration disorders in patients with MS mainly involve the motor and sensory spheres [7].

To date, few studies have been conducted in area of sensory integration disorders in patients with MS.

Stern et al. showed that individuals with higher sensory sensitivity, sensation avoidance and a low registration pattern had higher levels of anxiety compared to the general population, as well as lower mental and physical health-related quality of life. The pattern of sensation-seeking, on the other hand, showed the opposite characteristics, which the authors believe could suggest a potential protective factor in MS [19]. Hebert et al. showed that sensory integration disorders influenced postural disorders in patients with MS [20]. Another study observed that training targeting sensory integration disorders had a beneficial effect on balance disorders in MS patients, as well as on quality of life and improvements in their perceived fatigue [21,22].

As of today, the association between the occurrence of MSRF and depressive disorders in patients with MS seems fairly well documented, while little is known about the association of these disorders with somatosensory integration disorders. The association of somatosensory integration disorders with the course of MS has not yet been studied.

The purpose of this study is to demonstrate whether there is an association between somatosensory integration disorders in patients with relapsing–remitting MS and other features, such as: the occurrence of MS-related fatigue, depression and whether somatosensory integration disorders in patients with the relapsing–remitting form of MS is related to the duration and severity of the disease.

## 2. Materials and Methods

### 2.1. Study Design and Participants

This cross-sectional study included 205 adults (147 females, 58 males; age range 19–69 years) with a confirmed diagnosis of relapsing–remitting multiple sclerosis (RRMS). Patients were recruited consecutively from the National Health Insurance Drug Program at the Department of Neurology, Pomeranian Medical University in Szczecin, between January 2022 and March 2023.

Inclusion criteria

- Age ≥ 18 years,
- Confirmed diagnosis of RRMS,
- Active treatment within the NHI Drug Program,
- Clinical stability without relapse within 8 weeks prior to assessment.

Exclusion criteria

- Age < 18 years,
- Primary or secondary progressive MS,
- Relapse within the 8 weeks preceding evaluation.

All participants provided written informed consent. The study received an exemption from ethics board approval (KB.006.34.2023).

### 2.2. Assessment of Sensory Integration

Somatosensory integration was evaluated using the Daniel Travis Adult Sensory Integration Questionnaire (Supplementary Materials) [16], previously validated in the Polish population under supervision of certified sensory integration specialists. One of the authors is a certified Ayres Sensory Integration Therapist (ASI/SIAT).

The questionnaire comprises six domains:

1. General modulation (9 items);
2. Over-responsiveness (26 items);
3. Under-responsiveness/Sensory seeking (20 items);
4. Sensory discrimination (26 items);
5. Sensory-based motor abilities (19 items);
6. Social and emotional functioning (22 items).

Items are rated on a 0–4 Likert scale (0 = never; 4 = occurs regularly) [23]. Past but resolved symptoms are marked as “P.” Scores for each domain are summed; higher scores indicate greater sensory integration difficulties. Elevated scores are suggestive of SID but require clinical interpretation.

### 2.3. Assessment of Depression

Depressive symptoms were assessed using the Beck Depression Inventory (BDI) (Supplementary Materials) [24], a validated 21-item self-report scale. Each item is rated 0–3, producing a total score reflecting depression severity.

Patients were classified into the following subgroups:

- A: 0–11—no depression/depressed mood;
- B: 12–19—mild depression;
- C: 20–25—moderate depression;
- D: ≥26—severe depression.

### 2.4. Disability Assessment

Neurological disability was assessed using the Expanded Disability Status Scale (EDSS) (Supplementary Materials) [25]. The scale evaluates eight functional systems and ambulation, with scores ranging from 0 (normal examination) to 10 (death due to MS).

For analysis, patients were classified into:

- E: EDSS ≥ 3;
- F: EDSS < 3.

The EDSS cutoffs of ≤3 and >3 were selected because they represent clinically meaningful thresholds widely used in MS research and clinical practice. An EDSS score of 0–3 reflects minimal to mild disability, where neurological deficits are present but do not significantly impair motor function or independence in daily activities. Patients in this range are typically fully ambulatory without assistance, and deficits are often confined to one or two functional systems (e.g., sensory or visual). In contrast, an EDSS score > 3 indicates the onset of definite functional impairment, particularly in motor and cerebellar domains. The threshold around EDSS 3–3.5 is recognized as the point at which disability begins to affect gait, balance, coordination, and activities of daily living. This cutoff is frequently used in MS studies to distinguish between preserved versus impaired motor functioning and to identify individuals at higher risk of accumulating long-term disability [25,26,27,28].

### 2.5. Disease Duration

Disease duration was calculated from the date of RRMS diagnosis. Patients whose diagnostic delay exceeded one year from symptom onset were excluded. Subgroups were defined as:

- G: ≤5 years;
- H: 5–10 years;
- J: >10 years.

### 2.6. MSRF Assessment

MS-related fatigue (MSRF) was evaluated using the Fatigue Scale for Motor and Cognitive Functions (FSMC) (licensed for this study; agreement dated 25 April 2018 with Clinical Neuroscience Consulting GmbH). (Supplementary Materials) [29].

The FSMC includes 20 items rated on a 1–5 Likert scale and provides:

- A total fatigue score (0–100);
- Motor and cognitive fatigue subscores.

MSRF was defined as:

- FSMC ≥ 43 (mild: 43–52; moderate: 53–62; severe: ≥63).

Participants were classified as:

- MSRF present: total score ≥ 43;
- MSRF absent: total score < 43.

### 2.7. MRI Assessment of T2 Lesion Burden

For each patient, the most recent contrast-enhanced brain MRI was analyzed. Studies were conducted at the Department of Imaging Diagnostics and Interventional Radiology (USK 1 PUM, Szczecin) using a 1.5 T GE Discovery 450 scanner (GE Healthcare, Kraków, Poland).

The number of hyperintense lesions on T2-weighted sequences was categorized as:

- K: <10 lesions;
- L: 10–20 lesions;
- M: >20 lesions.

### 2.8. Disease Activity

Disease activity was defined as the number of clinical relapses within the previous 12 months. Subgroups were:

- N: 0 relapses;
- O: 1 relapse;
- P: ≥2 relapses.

### 2.9. Statistical Analysis

The test power and sample size calculated for two means—independent samples—reached values of N from 0.6 to 0.9. This allows us to analyze the material and draw reliable conclusions.

Continuous variables were summarized using means, standard deviations, medians, and quartiles; categorical variables using counts and percentages. Levene’s test indicated variance heterogeneity (*p* < 0.05), and Shapiro–Wilk tests showed non-normal distributions (*p* < 0.05). Therefore, non-parametric tests were applied:

- Mann–Whitney U test for two-group comparisons,
- Kruskal–Wallis ANOVA and median test for comparisons across ≥3 groups.

The significance threshold was *p* < 0.05. Analyses were performed using STATISTICA 13.3 (licensed version).

## 3. Results

Women predominated in the study group (71.70% vs. 28.29%). Most of the patients (61.16%) did not show symptoms of depression. In the study group, most patients had a disability level resulting from the disease according to the EDSS lower than 3 points (78.04% vs. 21.46%). The vast majority of patients experienced MS-related fatigue (65.85% vs. 34.14%). In the study group, most patients did not experience a relapse of the disease in the last year (76.097%).

Because the data violated normality (Shapiro–Wilk test, *p* < 0.05) and showed heterogeneity of variances (Levene’s test, *p* < 0.05), sensory integration scores between the two groups were compared using the Mann–Whitney U test. A two-tailed significance level of *p* < 0.05 was applied.

Table 1 presents SID in patients with relapsing–remitting MS who experienced mood swings without depressive disorders (subgroup A), with mild depressive disorders (subgroup B). Subgroups C (moderate depression) and D (severe depression) contained only 17 and 15 individuals, respectively. Due to insufficient statistical power, these subgroups were excluded from the main comparative analysis. The table therefore focuses on the comparison between patients with no depressive symptoms (A) and those with mild depressive symptoms (B), representing the only groups with adequate sample sizes for reliable non-parametric testing.

Statistically worse results were demonstrated in the following domains: hypersensitivity, sensory discrimination and social and emotional skills in subgroups diagnosed with depression compared to the subgroup without depression.

The Mann–Whitney U test was applied to compare the median differences between the two independent EDSS subgroups (≥3 vs. <3).

Table 2 presents SID in patients with MS who obtained an EDSS score for assessing the severity of disability of less than or equal to 3 (subgroup E) and above 3 (subgroup F).

Patients with a more severe degree of disability (scoring more than 3 points on the EDSS) had more severe sensory discrimination (*p* = 0.013 *) and motor function (*p* = 0.00 *) disorders.

*p*-values were calculated using the Kruskal–Wallis test due to non-normal data distribution and heterogeneity of variances.

Table 3 Shows SID and disease duration from 0 to 5 years (group G), from 5 to 10 years (subgroup H), and from 10 years (subgroup J) in patients with relapsing–remitting MS.

It should be noted that patients with a shorter disease duration (less than 5 years) have significantly better overall SI parameters compared to groups with a disease duration of more than 5 years (more than 5 years *p* = 0.038 * and more than 10 years *p* = 0.037 *). Of course, As the duration of the disease increases, patients have poorer motor function compared to those with shorter disease duration (over 10 years of disease *p* = 0.042 *), which is characteristic of the course of MS.

Patients with MS-related fatigue scored worse on the somatosensory integration assessment in each domain than patients without fatigue. However, the differences were not statistically significant (*p* > 0.5).

*p*-values were calculated using the Kruskal–Wallis test due to non-normal distribution and heterogeneity of variances.

Table 4 presents sensory integration disorders in patients who have not experienced a relapse of the disease during the last year (subgroup N), experienced one relapse during last year (subgroup O), and experienced two or more relapses during the last year (subgroup P).

There were statistically significantly worse results in the domains of under-responsiveness/sensory seeking (*p* = 0.039 *) and social and emotional abilities in the group of patients with one (*p* = 0.025 *) and two relapses (*p* = 0.007 *) in the last year compared to the group of patients without relapses.

No association was found between scores in any of the SID domains and the number of hyperintense lesions in the T2-weighted sequence in brain MRI.

## 4. Discussion

In the presented study, we examined the relationship between somatosensory integration disorders (SID) and key clinical characteristics of relapsing–remitting MS (RRMS), including MS-related fatigue (MSRF), depressive symptoms, disability level, disease duration, and disease activity. Although sensory hypersensitivity is well documented in MSRF and has been reported in up to 48–90% of individuals, data on sensory hypersensitivity in MS remain limited [30,31]. Previous studies indicate that abnormalities in sensory processing may contribute to symptom severity and functional impairment in MS [20,21]. Our findings expand this evidence by demonstrating that SIDs are closely associated with depressive symptoms, disability progression, and disease activity, but not with MSRF.

Patients with MSRF exhibited consistently higher SID scores across all domains; however, none of the differences reached statistical significance. This aligns with prior research suggesting that while sensory processing alterations may contribute to fatigue, they are not the sole or primary driver in RRMS [32].

The observed trend toward poorer SID scores in the MSRF group may reflect subtle disruptions in sensory integration that are not fully captured by questionnaire-based assessments or require larger sample sizes to detect. Notably, MS-related fatigue is increasingly recognized as a multifactorial phenomenon, influenced by neuroinflammatory processes, sleep disturbances, and neuropsychiatric comorbidities which may overshadow the contribution of sensory integration deficits. For instance, sleep disorders are prevalent in MS and have been shown to exacerbate fatigue independently of disease severity, suggesting that SID may represent only one component within a complex network of interacting mechanisms [33,34].

These findings underscore the need for multimodal approaches to understanding and treating MSRF, potentially combining objective sensory assessments with evaluations of sleep, mood, and inflammation.

A clear and significant association emerged between depressive symptoms and sensory integration dysfunction (SID). Patients with mild depression in our study exhibited greater sensory sensitivity, impaired sensory discrimination, and reduced social–emotional functioning compared with non-depressed patients. These findings are consistent with previous studies showing that depression is associated with altered sensory processing, including hyper-responsivity and deficits in perceptual filtering, which in turn affect attentional control and social functioning [35,36,37,38].

For example, hypersensitivity may lead to sensory overload, making everyday social and environmental interactions more challenging and potentially reinforcing feelings of withdrawal and low mood. Conversely, altered sensory processing may contribute to the development or persistence of depressive symptoms. By amplifying emotional distress or reducing adaptive coping capacities, sensory dysfunction can act as a vulnerability factor for depression [39,40,41].

This bidirectional relationship suggests that SID is not merely a consequence of depression but may play a mechanistic role in its onset and maintenance. Our results align with evidence indicating that interventions targeting sensory processing—such as sensory integration therapy or structured sensory modulation strategies—can improve emotional regulation and social participation in individuals with neurological conditions, including multiple sclerosis [42].

Overall, these findings underscore the clinical importance of considering sensory processing profiles in depressed patients with MS. Sensory-focused interventions may complement conventional pharmacological and psychological treatments, offering a multidimensional approach that addresses both the neurobiological and psychosocial aspects of depression. Future studies may explore longitudinal trajectories to better understand causality and identify which sensory interventions yield the most significant improvements in depressive symptoms and functional outcomes.

The observed associations between SID scores and clinical disability are consistent with previous research demonstrating that sensory processing impairments in multiple sclerosis (MS) are closely linked to disease severity. Patients with an Expanded Disability Status Scale (EDSS) score of ≥3 exhibited marked deficits in sensory discrimination, hypersensitivity, and motor-based sensory integration, highlighting the vulnerability of sensorimotor pathways commonly affected in MS (e.g., cortical and subcortical white matter lesions). These findings align with reports that longer disease duration, particularly exceeding 10 years, correlates with reduced motor performance and diminished general modulation of sensory input, reflecting cumulative neurodegenerative changes over time. Prior studies have underscored that both white-matter tract integrity and cortical network connectivity are crucial for maintaining normal sensory function, and progressive damage in these systems may underlie the sensory deficits observed in patients with more advanced MS [1,2,3,4,43,44,45].

Collectively, these results support the notion that sensory processing disturbances in MS are not only early markers of functional impairment but also evolve alongside structural neurodegeneration, emphasizing the importance of integrating sensory assessments into routine clinical evaluation.

Patients who experienced one or more clinical relapses in the previous year demonstrated significantly higher scores in under-responsiveness/sensory seeking and social–emotional dysfunction compared with relapse-free patients. This pattern suggests that recent inflammatory activity may transiently disrupt multisensory integration networks, or that greater disease instability exacerbates difficulties in emotional and perceptual regulation [45].

Notably, no relationship was observed between SID scores and T2 lesion count, indicating that functional impairments in MS may be more closely linked to network-level disruption than to total lesion burden. These findings align with evidence that social-cognitive and emotional processing deficits can manifest early in relapsing–remitting MS, even in patients with low disability and preserved global cognitive function.

Such impairments appear independent of overt structural damage, emphasizing the role of subtle microstructural or connectivity alterations in driving sensory and emotional dysfunction. Collectively, these results suggest the importance of assessing functional network integrity in MS, beyond conventional lesion metrics. Clinically, monitoring sensory integration and social–emotional function could provide sensitive indicators of disease activity and relapse-related exacerbation. Future interventions aimed at supporting network-level function, including targeted rehabilitation or neuromodulatory strategies, may complement conventional disease-modifying therapies to mitigate these functional deficits.

The observed associations emphasize the importance of integrating sensory processing assessment into routine neurological and neuropsychological evaluations. Clinical interventions such as sensory discrimination training, sensory modulation therapy, and motor-sensory integration rehabilitation may support emotional regulation, functional stability, and overall quality of life. Notably, randomized trials have shown that sensory integration-based balance training can improve postural control and functional outcomes in MS patients, supporting the clinical relevance of targeted sensory interventions [21].

However, several limitations should be considered. Many studies, including ours, are cross-sectional, limiting causal inference between SID and clinical outcomes. Sample sizes are often modest, potentially restricting generalizability [46,47,48,49,50].

In the primary analysis, results were reported without correction to preserve their clinical relevance and ensure full transparency. Given the moderate number of comparisons, the Holm correction was subsequently applied, which revealed that the worse general modulation scores observed in patients with longer disease duration did not reach statistical significance. However, all statistically significant findings were confirmed in the post hoc analysis using the Bonferroni correction.

Furthermore, there was a clear inequality in gender distribution—women significantly predominated in the study group. Moreover, the assessment of sensory abnormalities did not include objective measures (e.g., quantitative sensory testing, electrophysiology), which may detect subtler deficits. Additionally, heterogeneity in assessment methods and the subjective nature of sensory symptom reporting complicate direct comparisons across studies.

Finally, lesion count alone may not fully reflect structural correlates of SID; future studies incorporating lesion location, cortical thickness, and white-matter microstructure are warranted.

Future longitudinal and interventional studies are needed to clarify the temporal dynamics of SID in RRMS and to evaluate the efficacy of sensory-targeted therapies on both functional and psychological outcomes.

In conclusion, our study reinforces the clinical significance of SID in RRMS and underscores the value of incorporating sensory assessments into comprehensive MS care. Addressing sensory dysfunction may not only alleviate non-motor symptoms but also enhance emotional well-being, functional independence, and overall quality of life.

## 5. Conclusions

This study demonstrates that somatosensory integration disorders are significantly associated with depressive symptoms, greater disability, longer disease duration, and increased relapse activity in RRMS. No significant association was observed between SID and MSRF. These findings highlight the multidimensional nature of sensory processing disturbances in MS and underscore the importance of incorporating SID evaluation into comprehensive clinical assessment and therapeutic planning.

## Figures and Tables

**Table 1:** Sensory Integration Scores in Patients With and Without Mild Depression.

Sensory Integration Domain	Subgroup A: No Depression (n = 137) Mean ± SD	Subgroup B: Mild Depression (n = 36) Mean ± SD	*p*-Value
General Modulation	9.03 ± 6.11	11.81 ± 7.57	0.80
Over-Responsiveness	24.19 ± 17.56	30.94 ± 19.12	0.41
Under-Responsiveness/Sensory Seeking	18.51 ± 12.68	25.66 ± 10.99	0.04 *
Sensory Discrimination	14.97 ± 13.86	26.19 ± 18.77	0.02 *
Sensory-Based Motor Abilities	16.26 ± 12.55	25.47 ± 18.66	0.11
Social and Emotional Functioning	19.18 ± 13.47	32.78 ± 20.30	0.01 *

Statistically significant values (*p* < 0.05) are marked with an asterisk.

**Table 2:** Sensory integration disorders in patients with MS and EDSS score.

Sensory Integration Domain	EDSS ≥ 3 (n = 44) Mean ± SD	EDSS < 3 (n = 160) Mean ± SD	*p*-Value
General Modulation (points)	8.29 ± 6.48	9.98 ± 7.20	0.116
Over-Responsiveness (points)	20.57 ± 17.25	27.55 ± 20.63	0.054
Under-Responsiveness/Sensory Seeking (points)	18.84 ± 12.43	17.45 ± 12.51	0.454
Sensory Discrimination (points)	13.96 ± 14.71	23.09 ± 22.03	0.013 *
Sensory-Based Motor Abilities (points)	13.05 ± 13.16	25.55 ± 18.66	0.000 *
Social and Emotional Functioning (points)	18.99 ± 16.08	22.75 ± 19.24	0.289

Statistically significant values (*p* < 0.05) are marked with an asterisk.

**Table 3:** Sensory integration disorders and disease duration.

Variables	Subgroup G ≤ 5 Years (n = 60) Mean ± SD	Subgroup H 5–10 Years (n = 77) Mean ± SD	Subgroup J >10 Years (n = 68) Mean ± SD	*p*-Value
General Modulation (points)	6.92 ± 6.41	9.45 ± 6.94	9.17 ± 6.38	0.038 *
Over-Responsiveness (points)	17.95 ± 15.84	22.87 ± 18.09	24.49 ± 19.85	0.213
Under-Responsiveness/Sensory Seeking (points)	18.23 ± 12.99	19.09 ± 12.17	17.26 ± 12.21	0.589
Sensory Discrimination (points)	12.03 ± 13.68	16.22 ± 14.63	18.80 ± 20.90	0.232
Sensory-Based Motor Abilities (points)	11.20 ± 11.73	15.44 ± 13.26	19.88 ± 18.88	0.042 *
Social and Emotional Functioning (points)	17.32 ± 15.74	21.30 ± 16.91	20.06 ± 17.62	0.420

Statistically significant values (*p* < 0.05) are marked with an asterisk.

**Table 4:** Sensory integration disorders and disease activity.

Variables	Subgroup N 0 Relapses (n = 156) Mean ± SD	Subgroup O 1 Relapse (n = 34) Mean ± SD	Subgroup P ≥2 Relapses (n = 14) Mean ± SD	*p*-Value
General Modulation (points)	8.89 ± 6.94	7.24 ± 5.55	9.50 ± 5.76	0.783
Over-Responsiveness (points)	21.82 ± 18.50	21.00 ± 17.25	27.43 ± 17.45	0.496
Under-Responsiveness/Sensory Seeking (points)	17.99 ± 12.08	17.32 ± 12.12	27.64 ± 14.22	0.039 *
Sensory Discrimination (points)	15.87 ± 17.62	13.38 ± 13.27	22.57 ± 15.79	0.183
Sensory-Based Motor Abilities (points)	15.59 ± 15.98	13.74 ± 12.08	22.21 ± 14.42	0.107
Social and Emotional Functioning (points)	19.69 ± 17.05	15.21 ± 13.04	32.14 ± 17.47	0.025 *

Statistically significant values (*p* < 0.05) are marked with an asterisk.

## Data Availability

The data cannot be made publicly available due to privacy regulations.

## Supplementary Materials

The following supporting information can be downloaded at: https://www.mdpi.com/article/10.3390/jcm15010065/s1.
