# Supplementary material for: The Association Between the Occurrence of Sensory Integration Disorders, Depression, and Chronic Fatigue in Patients with Relapsing–Remitting Multiple Sclerosis
Source: J Clin Med. 2025 Dec 22;15(1):65. doi: 10.3390/jcm15010065 (PMC12786681; doi:10.3390/jcm15010065)
Supplement: Supplementary file 1 [file jcm-15-00065-s001.zip › jcm-3990178-supplementary/ExpandedDisabilityStatusScale(EDSS)_NeurologicalScales.pdf]

Expanded Disability Status Scale (EDSS)

The Expanded Disability Status Scale (EDSS) is a commonly used scale for assessing the level of disability in people with multiple sclerosis. The scale was originally described by Kurtze (1983) and has been adapted on the Multiple Sclerosis Trust website.

The *Criteria* requires that each of the functional systems listed below be reviewed. The description in the table below should be referred to and for ambulant patients a score from 0 (no disability) to 5.5 (more severe disability) be indicated. If the patient has impairment such that assistance is required to enable walking (cane, crutches, frame) the score will be from 0 to 6.5, if a wheelchair is required for all but a few steps the score will be 7.0 – 8.5, if confined to bed the score will be 9 and death due to MS is scored 10.

Functional systems

- pyramidal - weakness or difficulty moving limbs
- cerebellar - ataxia, loss of coordination or tremor
- brainstem - problems with speech, swallowing and nystagmus
- sensory - numbness or loss of sensations
- bowel and bladder function
- visual function
- cerebral (or mental) functions
- other

ALL functional system scores must be scored individually and then the EDSS calculated from the table below.

Expanded Disability Status Scale (EDSS)

Score	Description
0.0	No disability
1.0	No disability, minimal signs in one FS
1.5	No disability, minimal signs in more than one FS
2.0	Minimal disability in one FS
2.5	Mild disability in one FS or minimal disability in two FS
3.0	Moderate disability in one FS, or mild disability in three or four FS. No impairment to walking
3.5	Moderate disability in one FS and more than minimal disability in several others. No impairment to walking
4.0	Significant disability but self-sufficient and up and about some 12 hours a day. Able to walk without aid or rest for 500m
4.5	Significant disability but up and about much of the day, able to work a full day, may otherwise have some limitation of full activity or require minimal assistance. Able to walk without aid or rest for 300m
5.0	Disability severe enough to impair full daily activities and ability to work a full day without special provisions. Able to walk without aid or rest for 200m
5.5	Disability severe enough to preclude full daily activities. Able to walk without aid or rest for 100m
6.0	Requires a walking aid - cane, crutch, etc. - to walk about 100m with or without resting
6.5	Requires two walking aids - pair of canes, crutches, etc. - to walk about 20m without resting
7.0	Unable to walk beyond approximately 5m even with aid. Essentially restricted to wheelchair; though wheels self in standard wheelchair and transfers alone. Up and about in wheelchair some 12 hours a day
7.5	Unable to take more than a few steps. Restricted to wheelchair and may need aid in transferring. Can wheel self but cannot carry on in standard wheelchair for a full day and may require a motorised wheelchair
8.0	Essentially restricted to bed or chair or pushed in wheelchair. May be out of bed itself much of the day. Retains many self-care functions. Generally has effective use of arms
8.5	Essentially restricted to bed much of day. Has some effective use of arms retains some self-care functions
9.0	Confined to bed. Can still communicate and eat
9.5	Confined to bed and totally dependent. Unable to communicate effectively or eat/swallow
10.0	Death due to MS

The Expanded Disability Status Scale has been replicated from the Multiple Sclerosis Trust website at:

- https://www.mstrust.org.uk/a-z/expanded-disability-status-scale-edss with permission from the Multiple Sclerosis Trust UK
