# Supplementary material for: The Association Between the Occurrence of Sensory Integration Disorders, Depression, and Chronic Fatigue in Patients with Relapsing–Remitting Multiple Sclerosis
Source: J Clin Med. 2025 Dec 22;15(1):65. doi: 10.3390/jcm15010065 (PMC12786681; doi:10.3390/jcm15010065)
Supplement: Supplementary file 1 [file jcm-15-00065-s001.zip › jcm-3990178-supplementary/Questionnaire_Kappos_et_al.php1_.pdf]

FSMC

Fatigue Scale for Motor and Cognitive Functions

Date: ___________

ID: ___________

Initials: ______________________

Age: ______________________ Sex: m ◯ f ◯

Instructions

The following questionnaire is about problems in everyday life which are directly associated with an extreme form of tiredness (fatigue). This extreme form of tiredness refers to an overwhelming state of lethargy, exhaustion and lack of energy which comes on abruptly and is unrelated to any obvious external causes. It does not mean the sort of isolated episodes which everyone might experience in the course of the day, after exertion, or after a sleepless night!
Please read each statement carefully. Then decide to what extent each statement applies to you and your everyday life. Please try not to base your answers on the way you are feeling at the moment; instead try to give us a picture of the way you feel in normal day-to-day life. Please put a cross in the appropriate circle (only one cross per statement please!).

	Does not apply at all	Does not apply much	Slightly applies	Applies a lot	Applies completely
1. When I concentrate for a long time, I get exhausted sooner than other people of my age.	◯	◯	◯	◯	◯
2. When I am experiencing episodes of exhaustion, my movements become noticeably clumsier and less coordinated.	◯	◯	◯	◯	◯
3. Because of my episodes of exhaustion, I now need more frequent and/or longer rests during physical activity than I used to.	◯	◯	◯	◯	◯
4. When I am experiencing episodes of exhaustion, I am incapable of making decisions.	◯	◯	◯	◯	◯
5. When faced with stressful situations, I now find that I get physically exhausted quicker than I used to.	◯	◯	◯	◯	◯
6. Because of my episodes of exhaustion, I now have considerably less social contact than I used to.	◯	◯	◯	◯	◯
7. Because of my episodes of exhaustion, I now find it more difficult to learn new things than I used to.	◯	◯	◯	◯	◯

Please turn over

FSMC-cog = ______________ FSMC-mot = ______________ **FSMC total** = ______________

© *Penner et al., 2005*

	Does not apply at all	Does not apply much	Slightly applies	Applies a lot	Applies comple-tely
8. The demands of my work exhaust me mentally more quickly than they used to.	◯	◯	◯	◯	◯
9. I feel the episodes of exhaustion particularly strongly in my muscles.	◯	◯	◯	◯	◯
10. I no longer have the stamina for long periods of physical activity that I used to have.	◯	◯	◯	◯	◯
11. My powers of concentration decrease considerably when I'm under stress.	◯	◯	◯	◯	◯
12. When I am experiencing episodes of exhaustion, I am less motivated than others to start activities that involve physical effort.	◯	◯	◯	◯	◯
13. My thinking gets increasingly slow when it is hot.	◯	◯	◯	◯	◯
14. When I am experiencing an episode of exhaustion, my movements become noticeably slower.	◯	◯	◯	◯	◯
15. Because of my episodes of exhaustion, I now feel less like doing things which require concentration.	◯	◯	◯	◯	◯
16. When an episode of exhaustion comes on, I am simply no longer able to react quickly.	◯	◯	◯	◯	◯
17. When I am experiencing episodes of exhaustion, certain words simply escape me.	◯	◯	◯	◯	◯
18. When I am experiencing episodes of exhaustion, I lose concentration considerably quicker than I used to.	◯	◯	◯	◯	◯
19. When it is hot, my main feeling is one of extreme physical weakness and lack of energy.	◯	◯	◯	◯	◯
20. During episodes of exhaustion, I am noticeably more forgetful.	◯	◯	◯	◯	◯

Please make sure that you have written down your initials, age and sex on page 1 and have put a cross by each statement. Thank you.

© Penner et al., 2005
